# Post-translational modifications in extracellular microvesicles from plasma of rheumatoid arthritis patients following anti-TNFα therapy

**DOI:** 10.1093/cei/uxag044

**Published:** 2026-08-01

**Authors:** Serena Recalchi, Gloria Riitano, Federica Maria Ucci, Valeria Manganelli, Tina Garofalo, Federica Fratini, Roberta Misasi, Agostina Longo, Cristiano Alessandri, Fabrizio Conti, Antonella Capozzi, Maurizio Sorice

**Affiliations:** Department of Experimental Medicine, ‘Sapienza’ University of Rome, Rome, Italy; Department of Life, Health and Health Professions Sciences, ‘Link Campus’ University, Rome, Italy; Department of Experimental Medicine, ‘Sapienza’ University of Rome, Rome, Italy; Rheumatology, Department of Clinical Internal, Anesthesiological and Cardiovascular Sciences, Sapienza University, Rome, Italy; Department of Experimental Medicine, ‘Sapienza’ University of Rome, Rome, Italy; Department of Wellbeing, Health and Environmental Sustainability, Sapienza University of Rome, Rome Italy; Department of Neurosciences, Istituto Superiore di Sanità, Rome, Italy; Department of Experimental Medicine, ‘Sapienza’ University of Rome, Rome, Italy; Department of Wellbeing, Health and Environmental Sustainability, Sapienza University of Rome, Rome Italy; Rheumatology, Department of Clinical Internal, Anesthesiological and Cardiovascular Sciences, Sapienza University, Rome, Italy; Department of Wellbeing, Health and Environmental Sustainability, Sapienza University of Rome, Rome Italy; Rheumatology, Department of Clinical Internal, Anesthesiological and Cardiovascular Sciences, Sapienza University, Rome, Italy; Department of Experimental Medicine, ‘Sapienza’ University of Rome, Rome, Italy; Department of Experimental Medicine, ‘Sapienza’ University of Rome, Rome, Italy

**Keywords:** rheumatoid arthritis, extracellular vesicles, autoantibodies, autoantigens, biomarkers, post-translational modified proteins

## Abstract

Autoantibodies targeting post-translational modified proteins represent key immunological hallmarks of Rheumatoid Arthritis (RA). In a previous study we demonstrated the presence of citrullinated and carbamylated proteins in extracellular microvesicles (EMVs) from plasma of RA patients. Since the pivotal role of TNFα in sustaining inflammatory and oxidative processes that promote protein modification, in this study we investigated the patterns of these post-translational modifications in EMVs isolated from RA patients before and after anti-TNFα treatment. Twelve consecutive RA patients, naïve to biological therapy, were enrolled and subjected to anti-TNFα therapy, which remained stable throughout the 8-month follow-up. EMVs, isolated from baseline (T0) and 8 months of follow-up (T8) plasma of RA patients, were measured by Nanoparticle Tracking Analysis or lysed and subjected to western blot analysis, using anti-citrulline or anti-carbamyl lysine antibodies. Anti-citrullinated and anti-carbamylated protein antibodies were also detected. Anti-TNFα treatment induced a significant reduction in the concentration of EMVs isolated from plasma of RA patients after 8 months of anti-TNFα treatment (*P* < 0.01). Moreover, in EMVs extracts citrullinated and carbamylated protein levels were significantly lower at T8, as compared to T0 (*P* < 0.01). Anti-TNFα treatment also induced a significant decrease of both anti-citrullinated and anti-carbamylated protein antibody levels, as well as of activity scores (SDAI and DAS28). These findings suggest the role of EMVs, as well as of citrullinated and carbamylated protein levels, as potential indicators of RA activity.

## Teaser

- In RA patients anti-TNFa therapy significantly reduces the concentration of circulating EMVs.
- In RA patients anti TNFa therapy significantly reduces citrullinated and carbamylated proteins, anti-citrullinated protein antibodies (ACPA) and anti-carbamylated protein (anti-CarP) antibodies.
- EMV-associated citrullinated and carbamylated proteins could represent novel biomarkers for monitoring therapeutic response and immune resolution in RA.

## Graphical Abstract

**Graphical Abstract:**
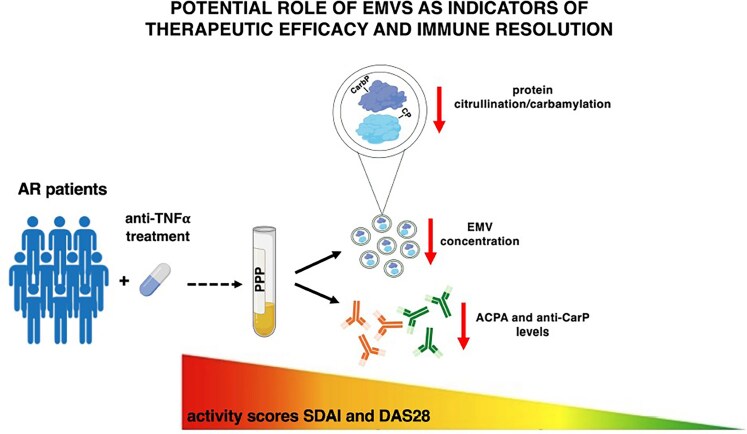

## Introduction

Rheumatoid arthritis (RA) is a systemic autoimmune disease characterized by chronic synovial inflammation, primarily affecting diarthrodial joints, leading to cartilage destruction and bone erosion. The etiology of RA is heterogeneous, involving intricate interactions among genetic predisposition, environmental factors, and dysregulated immune responses, affecting approximately 0.5–1% of the global population [1].

Autoantibodies targeting post-translational modified proteins have long been recognized as key immunological hallmarks of RA [2]. Rheumatoid factor (RF), first described in 1940, represents a heterogeneous group of antibodies directed against the Fc portion of IgG, with IgM isotype [3, 4]. The identification of anti-citrullinated protein antibodies (ACPA) further refined RA serology, providing both higher diagnostic specificity than RF and the ability to predict disease onset years before clinical manifestation [5]. Citrullinated and homocitrullinated proteins are abundant within the synovium and serum of seropositive patients, supporting the concept of a “RA citrullinome” [6]. More recently, anti-carbamylated protein antibodies (anti-CarP) have been identified as additional RA-related autoantibodies recognizing antigenic targets, such as fibrinogen, vimentin, and α-enolase [7]. Despite partial overlap with ACPA, anti-CarP antibodies represent distinct entities, detectable in 8–16% of ACPA-negative patients and correlating with greater disease activity and disability [8–10].

To standardize diagnosis, the 2010 ACR/EULAR criteria integrated serological, clinical, and inflammatory parameters, including RF/ACPA titers, erythrocyte sedimentation rate, C-reactive protein (CRP), joint involvement and symptom duration, to classify RA above a 6/10 threshold [11].

Disease activity and functional impairment in RA are routinely assessed using clinometric indices, including composite scores and patient-reported outcome measures, such as the Disease Activity Score (DAS28) on 28 joints and the Simplified Disease Activity Index (SDAI). Together with patient-reported outcomes, these tools offer a multidimensional evaluation of RA severity and functional impact [12–14].

Therapeutic approaches have progressively shifted toward promoting disease remission by targeting the mechanisms involved in disease progression. Conventional synthetic disease-modifying anti-rheumatic drugs (DMARDs), such as methotrexate remains first-line therapy [15, 16] while biologic DMARDs (bDMARDs) targeting key cytokines and immune cells, including TNF inhibitors, IL-6 receptor blockers, and B- or T-cell modulators, have revolutionized disease management [12]. More recently, targeted synthetic DMARDs (DMARDs), particularly Janus kinase inhibitors have offered oral therapeutic alternatives that interfere with intracellular cytokine signaling pathways [16, 17]. Overall, these advances reflect the transition of RA management toward a mechanism-based approach grounded in immunopathogenic understanding.

Elevated circulating levels of extracellular vesicle (EVs) and alterations in their molecular cargo have been reported as potential biomarkers in several autoimmune conditions, including RA [18]. EVs are spherical, lipid-bilayer delimited structures that are universally secreted by nearly all cell types and organisms. Based on characteristics, such as function, molecular cargo, size, and mode of secretion, EVs are generally classified into three main groups: exosomes (30–150 nm) formed through the inward budding of the multivesicular body, microvesicles (MVs, also called extracellular microvesicles, EMVs), which are generally 100–1000 nm in diameter and formed through outward budding of the plasma membrane and apoptotic bodies (>1000 nm), generated by plasma membrane blebbing of cells undergoing apoptosis. EVs represent a novel axis of intercellular communication, contributing not only to tissue homeostasis, but also to the pathogenesis of immune-mediated diseases [19, 20]. In autoimmune diseases, by shuttling bioactive molecules, including proteins, lipids, and nucleic acids, EVs may facilitate antigen presentation and amplify systemic inflammation. Emerging evidence indicates that EVs, mainly EMVs, act as active carriers of autoantigens and modified proteins, contributing to immune activation and joint inflammation in RA. Notably, citrullinated and carbamylated antigens have been detected on the surface of circulating EVs, suggesting that these vesicles may represent dynamic biomarkers of autoimmunity and treatment response [21–23].

Given the pivotal role of TNFα in sustaining inflammatory and oxidative processes that promote protein modification, anti-TNFα therapy might modulate the extent of citrullination and carbamylation at the EVs level. In this study, we investigated the patterns of these post-translational modifications in EMVs isolated from RA patients before and after TNFα inhibition, aiming to elucidate their role as potential indicators of RA activity.

## Materials and methods

### Patients

Twelve consecutive patients with RA, naïve to biological therapy, satisfying the 2010 ACR classification criteria for RA [11], were enrolled from the Arthritis Center of Sapienza University of Rome. The female-to-male ratio was 9:3, with a median age of 52.5 ± 17 years and a median disease duration of 36 ± 50.1 months. ACPA positivity was observed in 58.3% of patients, RF positivity in 50%, and 41.6% were positive for both autoantibodies. Smoking history was reported in 16.7% of patients. Two patients had concomitant hypertension and diabetes, and one patient had dyslipidemia. No patients had pulmonary disease or osteoporosis. At baseline, the median CRP level was 1.1 ± 3.1 mg/L. Median disease activity, assessed by SDAI and DAS28, was 20.3 ± 12.2 and 4.2 ± 1.4, respectively (Table 1). Anti-TNFα therapy with adalimumab (40 mg/2 weeks) remained stable throughout the 8 months of follow-up in all patients. The study protocol was approved by the Ethics Committee of Sapienza University of Rome (protocol no. 109/18), and the written informed consent was obtained from all participants prior to enrollment.

**Table 1:** Clinical characteristics of RA patients at baseline.

	RA patients (n = 12)
*Disease activity*	*Median (CI)*
Disease duration (months)^a^	36 (0.75–63)
PGA (0–100)^a^	55.42 (47.4–63.4)
DAS28^a^	3.97 (2.85–4.46)
SDAI^a^	20.28 (10.89–23.91)
*Laboratory parameters*	*n (%)*
RF	6 (50)
ACPA	7 (58.3)
*Features*	*n (%)*
Female	9 (75)
Male	3 (25)
Age in years^a^	52.5 (39.75–62.5)
Dyslipidemia	1 (8.3)
Hypertension	2 (16.7)
Diabetes (type 2)	2 (16.7)
Smoking Habit	2 (16.7)
*Treatments*	*n (%)*
No treatment	3 (25)
cDMARDs	8 (66.7)
*Glucocorticoids*	4 (33.3)

^a^Median (CI); PGA, Patient Global Assessment; DAS28, Disease Activity Score-28; SDAI, Simplified Disease Activity Index; RF, rheumatoid factor; ACPA, anti-citrullinated protein antibodies; cDMARDs, conventional synthetic disease-modifying anti-rheumatic drugs CRP, C-reactive protein.

### Detection of ACPA and anti-CarP

ACPA titers were tested using QUANTA Flash CCP chemiluminescence immunoassay (Werfen, San Diego, CA, USA), a commercial kit that specifically uses cyclic citrullinated peptides as antigens to capture the antibodies. Anti-CarP antibodies were detected by a commercial ELISA kit (AFG Bioscience, Northbrook, IL, USA). Both autoantibody assays were carried out in triplicate on all patient sera, and the results were evaluated according to the manufacturers’ instructions.

### Isolation of EMVs

Peripheral blood samples were collected from 12 RA patients at baseline (T0) and after 8 months of anti-TNFα treatment (T8), by venipuncture into 5-ml tubes containing sodium citrate. To obtain platelet-poor plasma, samples were centrifuged twice at ×2500 *g* for 15 min at room temperature. The resulting supernatant was transferred to new tubes and diluted with phosphate-buffered saline (PBS) to maintain stability during subsequent centrifugation. Samples were then centrifuged at ×14 000 g for 35 min at 4°C to isolate the EMVs fraction. The resulting pellets were washed once in PBS and finally resuspended in PBS for further analyses.

### Nanoparticle tracking analysis

Nanoparticle Tracking Analysis was performed using the NanoSight NS300 system (Malvern Panalytical, Malvern, UK) to determine the size distribution and concentration of EMVs isolated from patients with RA. The instrument was equipped with a 488 nm (blue) laser, a high sensitivity sCMOS camera, and an automated syringe pump. Sample flow was maintained at a syringe pump speed of 30 arbitrary units, corresponding to the relative dispensing rate of the system. Measurements were performed at 20°C under automated script control and five 60-s videos were recorded per sample. Video capture and data analysis were conducted using NTA 3.4 Build 3.4.4 software.

### Transmission electron microscopy

For transmission electron microscopy (TEM) analysis, EMVs were first washed with PBS and then fixed in 1% paraformaldehyde for 1 h at 4°C to preserve vesicle morphology. Post-fixation was performed using 1% osmium tetroxide in Veronal acetate buffer (pH 7.4) for 2 h at 4°C, enhancing membrane contrast and structural preservation.

Following fixation, EMVs were stained with uranyl acetate (5 mg/ml), dehydrated through a graded acetone series, and subsequently embedded in Epon 812 resin. Samples were examined using a transmission electron microscope (Zeiss, Oberkochen, Germany) for morphological assessment.

### Western blot analysis

EMVs isolated from plasma of 12 RA patients at T0 and T8 were lysed in RIPA buffer (100 mM NaCl, 1 mM EDTA, 1% Triton X-100, 10 mM Tris-HCl pH 7.4, 0.5% sodium deoxycholate, 0.1% SDS, Na_3_VO_4_) supplemented with a protease inhibitor cocktail (Sigma, Milan, Italy). Lysates were centrifuged at ×15 000 *g* for 15 min at 4°C to collect soluble proteins. Protein content was quantified using the Bradford method (Bio-Rad, Segrate, MI, Italy), and equal amounts of lysate were resolved on 10% SDS–polyacrylamide gels. Subsequently, proteins were transferred to polyvinylidene difluoride membranes (Bio-Rad).

Membranes were blocked with 5% non-fat dry milk prepared in tris-buffered saline, containing 0.05% Tween-20 and incubated with rabbit anti-citrulline (Millipore, Billerica, MA, USA), rabbit anti-carbamyl lysine (CliniSciences, Nanterre, France), or rabbit anti-β-tubulin (Abcam, Cambridge, UK). Detection was performed using horseradish peroxidase–conjugated anti-rabbit or anti-mouse secondary antibodies (Sigma). Immunoreactive bands were visualized through chemiluminescence, using the Clarity Western ECL detection system (Bio-Rad). Densitometric analysis was carried out with NIH ImageJ software (version 1.62, Mac OS X, Apple Computer International, Cupertino, CA, USA).

### Cytofluorimetric analysis of EMVs surface expression of citrullinated/carbamylated antigens

Flow cytometric analysis of EMVs was performed using a CytoFLEX cytometer (Beckman Coulter, Brea, CA, USA), optimized for small particles and equipped with a 405 nm violet laser. The gating strategy (FSC-A vs. SSC-A plot) was defined using FITC fluorescence intensity on the x-axis and Violet SSC-A on the y-axis, with the threshold set on the Violet SSC channel, and including the individual gates established with calibration beads. Size calibration and gate definition were performed using fluorescent Megamix-Plus SSC and Megamix-Plus FSC beads (100–900 nm), allowing the identification of particle populations within the expected vesicle size range (Fig. 3a).

To evaluate the surface expression of citrullinated/carbamylated antigens, EMVs were incubated with unconjugated antibodies to anti-citrullinated (Millipore, Billerica) and carbamylated proteins (CliniSciences) for 40 min at room temperature in PBS without calcium and magnesium. After a centrifugation at ×14 000 *g* for 30 min at 4°C, a secondary antibody FITC-conjugated (Sigma) was added for 30 min at room temperature in the dark, followed by a second centrifugation at ×14 000 *g* for 30 min at 4°C and acquired by flow cytometry.

### Statistical analysis

All data were verified in at least three different experiments and the statistical analyses were performed by GraphPad Prism 8.0 software Inc. (San Diego, CA, USA). *P*-values for all graphs were generated using Student's *t*-test or Wilcoxon test, as indicated in the figure legends (*P ≤ 0.05, **P ≤ 0.01, ***P ≤ 0.001).

## Results

### Quantitative analysis of plasma-derived EMVs in RA patients before and after anti-TNFα therapy

In this pilot study, we enrolled 12 patients with RA naïve to biological therapy, fulfilling the 2010 ACR criteria [11]. Anti-TNFα therapy remained stable throughout the 8 months of follow-up in all the patients.

Quantitative analysis of EMVs isolated from the plasma of the patients at baseline (T0) and after anti-TNFα therapy (T8) was performed by NanoSight, allowing determination of vesicle concentration and size distribution.

**Figure 1:**
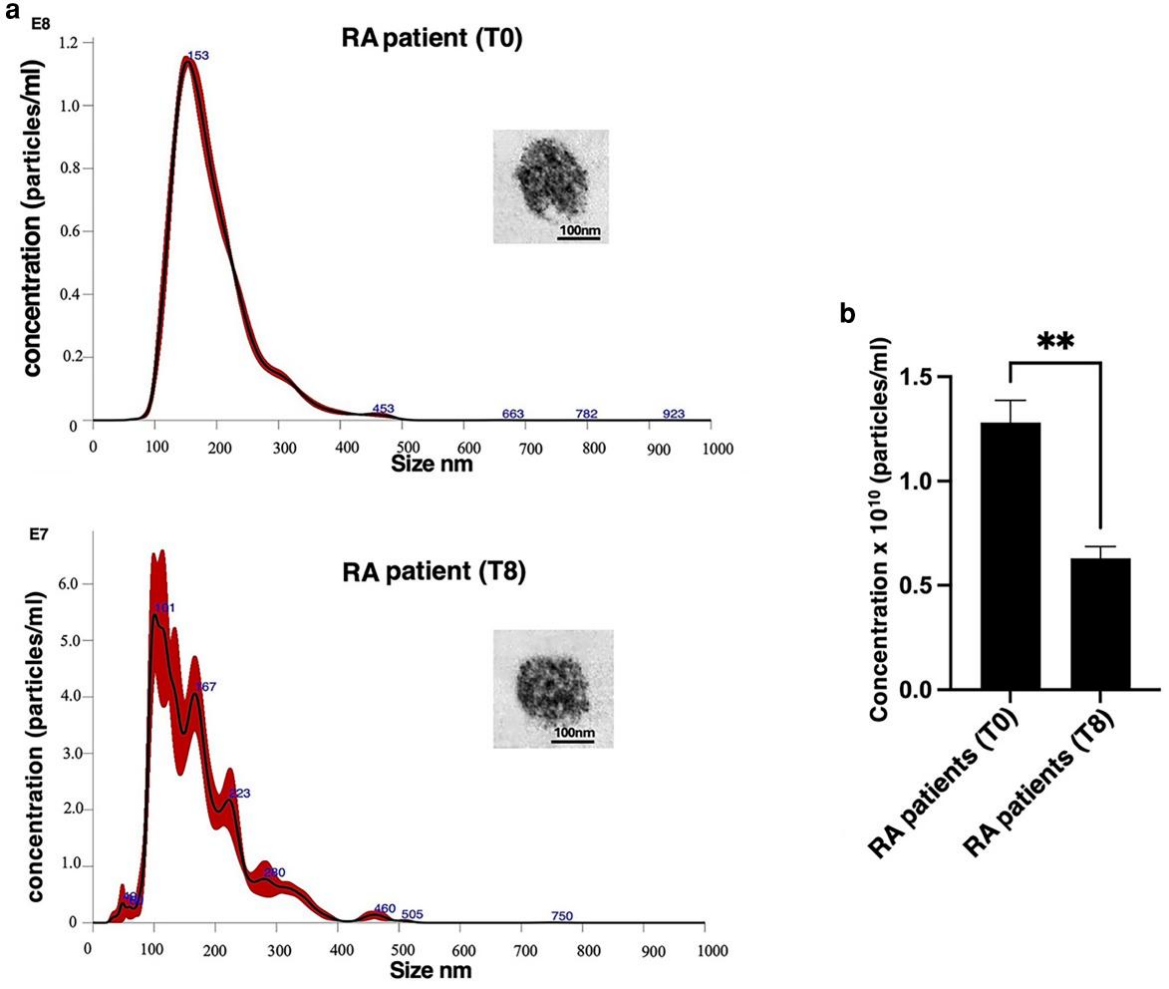
Quantitative analysis of EMVs from plasma of patients with RA before and after anti-TNFα treatment. EMVs isolated from one representative RA patient at baseline (T0) and after 8 months of treatment with TNFα inhibitors (T8) were analyzed by Nanoparticle Tracking Analysis (NanoSight) (A). Quantitative analysis showed that the number of EMVs in RA patients at baseline (T0) was 1.35 × 10^10^ EMVs/ml (SD 0.11 × 10^10^), compared to 0.63 × 10^10^ EMVs/ml (SD 0.06 × 10^10^) after TNFα inhibitor treatment (T8). ** *P* < 0.01 (B). Inserts: TEM analysis of EMVs.

At baseline (T0), patients exhibited elevated levels of circulating EMVs (1.35 × 10^10^ EMVs/ml; SD 0.11 × 10^10^). Following anti-TNFα treatment (T8), a significant reduction in EMV concentration was observed (0.63 × 10^10^ EMVs/ml; SD 0.06 × 10^10^) compared to baseline values (Figs. 1a and b), indicating that anti-TNFα is associated with a significant decrease in vesicle release (*P* < 0.01). This reduction suggests a modulation of vesicle shedding in response to the attenuation of systemic inflammation following anti-TNFα treatment. On the contrary, NanoSight analysis did not show a significant reduction of the mean size of EMVs after treatment with anti-TNFα (191.6 ± 1.3 nm vs. 171.9 ± 3.4 nm).

### Decrease of citrullinated and carbamylated protein levels in EMVs from plasma of patients with RA following anti-TNFα treatment

To investigate the presence of post-translationally modified proteins, EMVs samples isolated from all plasma of RA patients were lysed in RIPA buffer and subjected to western blot analysis, using anti-citrulline and anti-carbamyl lysine polyclonal antibodies.

**Figure 2:**
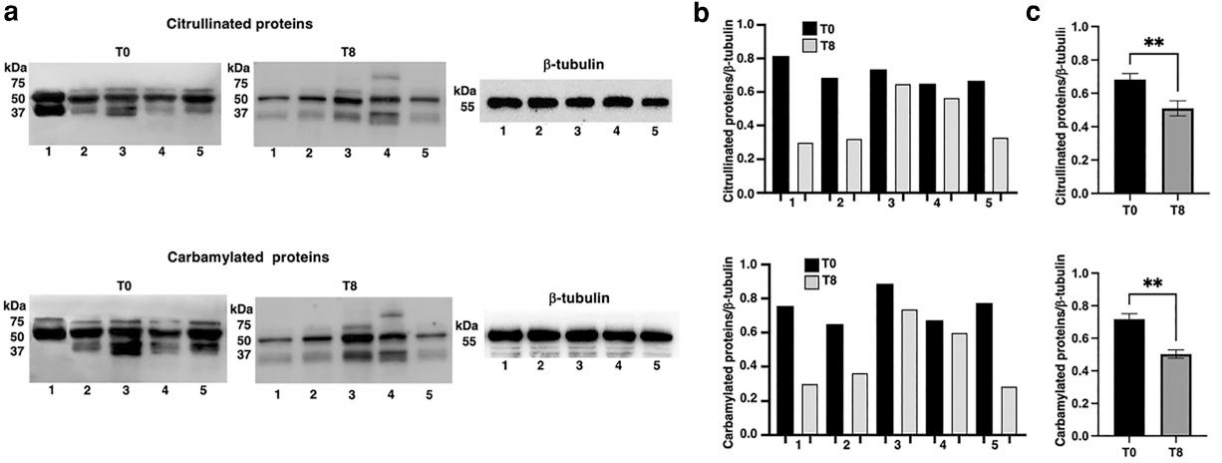
Analysis of post-translational modification levels in EMVs from RA patients following anti-TNFα treatment by Western blot. EMVs, isolated from RA patients at baseline (T0) and after 8 months of treatment with TNFα inhibitors (T8), were analyzed by western blot using anti-citrulline or anti-carbamyl lysine antibodies. Loading control was evaluated using anti-β-tubulin antibody. Western blot (a) and densitometric analysis (b) from 5 representative RA patients are shown. Densitometric values, calculated in all RA patients (c). Data are reported as mean (SD). *P*-values were generated using Wilcoxon's test. **P < 0.01.

**Figure 3:**
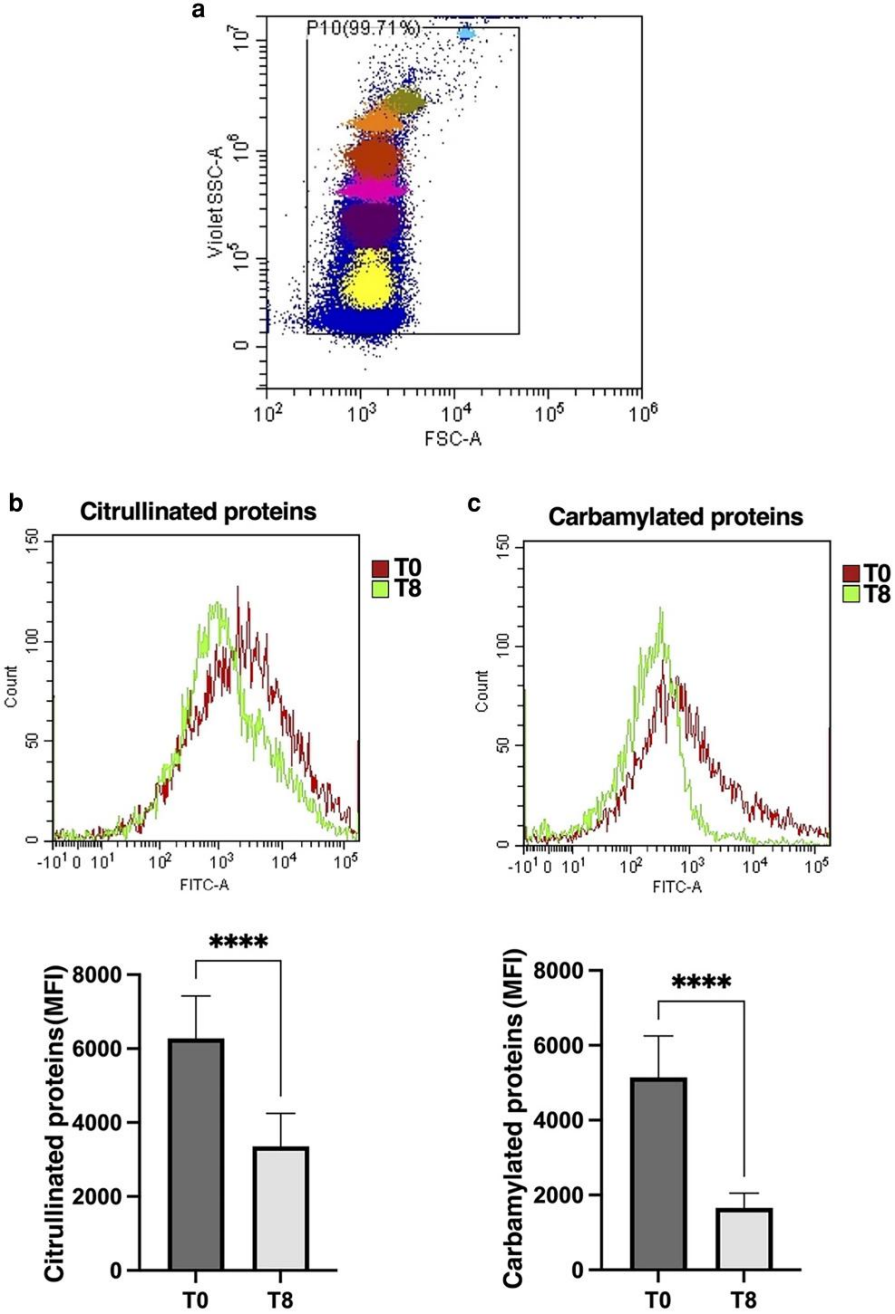
Analysis of post-translational modification levels in EMVs from RA patients following anti-TNFα treatment by flow cytometry. Representative FSC-A vs. violet SSC (vSSC) dot plot illustrating the gating strategy used to identify extracellular microvesicles (EMVs) isolated from patients with RA (a). The EMV population was defined based on forward scatter area (FSC-A) and violet side scatter (vSSC) characteristics using Megamix-Plus FSC and SSC fluorescent calibration beads (Beckman Coulter; 100–900 nm) to establish the size gate. Representative semiquantitative flow cytometry histogram overlays illustrating the decrease in surface expression of citrullinated (b) and carbamylated (c) proteins on EMVs isolated from patients with RA at week 8 (T8) compared to baseline (T0). Values were calculated in all RA patients. Data are reported as mean (SD). *P*-values were generated using Wilcoxon's test. ****P < 0.0001.

As shown in Fig. 2a, the intensity of immunoreactive bands was significantly decreased in EMVs isolated from RA patients after anti-TNFα therapy (T8) compared with baseline (T0). Five representative patients are shown in Figs. 2a and b. The densitometric analysis, including all RA patients (Fig. 1c), indicates a significant reduction in protein citrullination/carbamylation following treatment (*P* < 0.01). These results were confirmed by flow cytometry, which demonstrated a significant decrease of both anti-citrulline (Fig. 3b) and anti-carbamyl lysine (Fig. 3c) staining on the surface of EMVs (*P* < 0.0001). Overall, these findings indicate that anti-TNFα treatment is associated with a decrease in post-translational protein modifications carried by circulating EMVs, suggesting that the therapy may reduce the availability of modified autoantigens potentially involved in sustaining autoimmune responses in RA.

### Detection of anti-citrullinated and anti-carbamylated protein antibodies following anti-TNFα treatment

**Figure 4:**
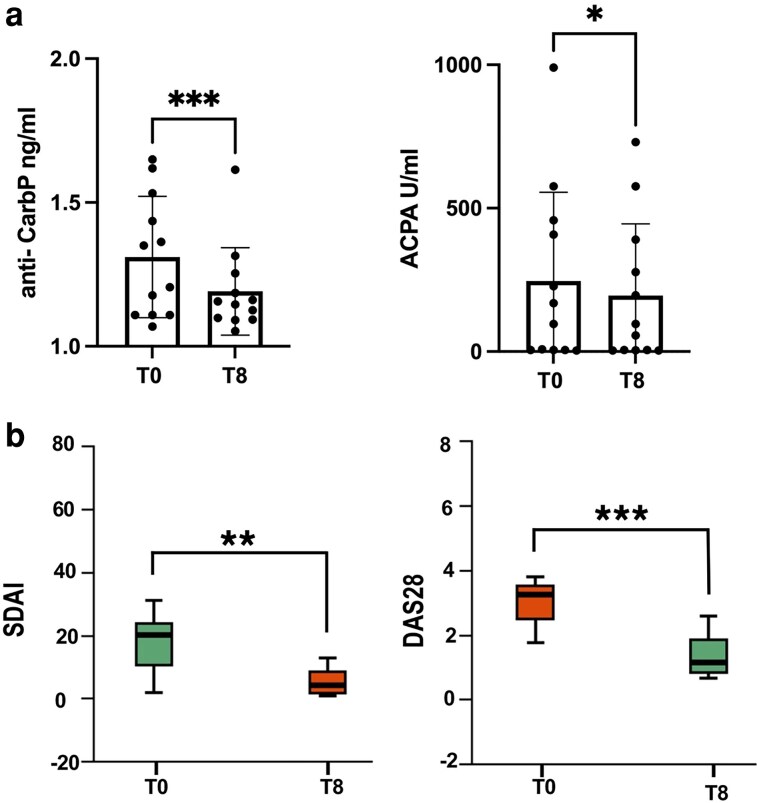
Effect of anti-TNFα therapy on anti-citrullinated and anti-carbamylated protein antibody levels and disease activity scores. The graphs show the effect of anti-TNFα treatment (T8) on ACPA and anti-CarbP antibody titers in RA patients’ sera (a) and disease activity scores (SDAI and DAS28) (b), compared to baseline (T0). *P*-values were generated using Wilcoxon's test. **P < 0.01, ***P < 0.001.

We analyzed whether treatment with anti-TNFα also modified antibody levels directed against citrullinated and carbamylated proteins (Fig. 4a). Indeed, ACPA and anti-CarP are well-recognized markers of autoimmune activation and are frequently associated with disease activity and progression. Quantitative analysis revealed that, on average, ACPA levels were significantly reduced at T8 compared to baseline (T0) (*P* < 0.05), as detected by chemiluminescence; accordingly, anti-CarP antibodies were significantly reduced (*P* < 0.001), as detected by ELISA. The reduction in ACPA and anti-CarP levels suggests that anti-TNFα treatment may not only attenuate inflammatory pathways but could also influence the autoimmune response associated with the disease.

### Evaluation of disease activity scores before and after anti-TNFα therapy

We analyzed whether treatment with anti-TNFα also modify activity scores of RA patients. After 8 months of anti-TNFα treatment, a significant reduction of SDAI from 20.3 ± 12.2 to 4.3 ± 4.3 (*P* < 0.01) and of DAS28 from 4.2 ± 1.4 to 1.7 ± 0.8 (*P* < 0.001) was observed (Fig. 4b), indicating that reduction of citrullinated and carbamylated protein levels in EMVs was accompanied by an improvement of inflammatory and clinical disease activity scores in RA patients receiving anti-TNFα therapy.

## Discussion

Our previous work demonstrated an increase of EMVs in the plasma of RA patients compared with healthy donors. In previous studies, we demonstrated that the main EMVs cellular sources are endothelium, leukocytes, and platelets [21]. EMVs exposing citrullinated and/or carbamylated antigens on their surface may act as unconventional antigen-presenting entities, stimulating T-cell responses and providing a continuous source of autoantigens that sustain chronic inflammation [24]. Moreover, key autoantigens in RA—such as vimentin, α-enolase, and type II collagen—were significantly enriched on EMVs from RA patients compared with healthy subjects. Expression of citrullinated determinants on EMV surfaces correlated positively with disease activity indices, suggesting a functional relationship between EV-associated protein modifications and clinical disease status [21].

In the present study, we demonstrate for the first time both a significant reduction of circulating EMVs and a decrease of their levels of citrullinated and carbamylated proteins in patients with RA, following 8 months therapy with anti-TNF-α. However, we cannot exclude the possibility that EVs modification may reflect overall suppression of inflammation. Indeed, this molecular alteration is accompanied by a reduction in circulating autoantibody titers—both anti-CarP antibodies and ACPA—as well as a concomitant improvement in clinical disease activity, as revealed by a significant decrease of SDAI and DAS28. However, although we observe an evident trend, correlation analyses do not reach statistical significance, probably due to the quite low number of patients under test.

These findings support that attenuation of post-translational-modification-driven neoantigen may play a role in mediating the immunological and clinical improvements associated with TNF-α inhibition. Our results are consistent with recent evidence indicating that EVs enriched in citrullinated and carbamylated antigens contribute to RA progression [21], suggesting that TNF-α inhibition not only suppresses inflammation, but may also reduce the generation of modified self-proteins via EMVs.

Some citrullinated molecules present on EVs can act as autoantigens, facilitating the formation of immune complexes, leading to monocyte activation and release of IL-1β, IL-6, and TNF-α. EVs, especially synovial ones, can also contribute to cartilage degradation and joint changes [25]. By shuttling bioactive molecules—including proteins, lipids, and nucleic acids—EVs may facilitate antigen presentation and amplify systemic inflammation. Elevated circulating levels of EVs and alterations in their molecular cargo have been reported as potential biomarkers in several autoimmune conditions, including RA [19]. Synovial fibroblast-derived EVs have been shown to carry citrullinated antigens capable of activating autoreactive T lymphocytes, thereby sustaining the autoimmune cascade [26].

These findings are also in line with literature data, indicating that TNFα inhibition may impair B-cell activation and autoantibody production through the restoration of immune homeostasis and reduction of inflammatory stimuli sustaining epitope spreading [27].

Clinically, reductions in ACPA and in anti-CarP titers have been documented following TNF-inhibitor therapy in some cohorts, supporting a role for TNF-α in sustaining epitope-spreading and peripheral autoantibody production [28].

This study is focused on a selected homogeneous cohort of RA patients treated with anti-TNFα therapy, but the main limitations are represented by the very small sample size and the absence of different groups of patients receiving alternative therapies.

Future studies are necessary to analyze whether these biomolecular changes precede and predict long-term joint protection and remission-sustenance, and whether quantification of EMV-bound citrullinated/carbamylated proteins could serve as novel biomarkers of therapeutic response.

## Data Availability

The data underlying this article will be shared on reasonable request to the corresponding author.
